# The Potential of Tele‐Ultrasound, Handheld and Self‐Operated Ultrasound in Pregnancy Care: A Systematic Review

**DOI:** 10.1002/pd.6679

**Published:** 2024-10-10

**Authors:** Shariva S. Kariman, Josephus F. M. van den Heuvel, Bauke M. E. Adriaanse, Dick Oepkes, Mireille N. Bekker

**Affiliations:** ^1^ Division of Obstetrics & Gynecology University Medical Center Utrecht Utrecht University Utrecht The Netherlands; ^2^ Division of Obstetrics Leiden University Medical Center Leiden The Netherlands

## Abstract

**Objective:**

To explore the use of tele‐ultrasound and handheld or self‐operated ultrasound in pregnancy.

**Methods:**

A systematic search provided 31 studies. The risk of bias for each study was assessed. Results were analyzed and presented in a narrative overview in four domains: tele‐ultrasound, patient‐operated ultrasound, handheld devices and low‐ and middle‐income countries (LMIC).

**Results:**

The quality of studies was generally low or fair based on the NIH Quality Assessment Tools. Fetal tele‐ultrasound services (11 studies) are feasible and especially helpful in rural areas or with increased centralization of specialist care. Three studies with patient‐operated ultrasound concluded its feasibility with good‐to‐high experiences. The use of handheld devices in pregnancy (eight studies) showed similar ultrasound results when compared to standard devices. In LMICs, innovative use of ultrasound (nine studies) can facilitate access to obstetric care performed by trained as well as unskilled caregivers combined with remote evaluation by an expert.

**Conclusions:**

Innovations in ultrasound in pregnancy care have shown promising results for application. Although most studies demonstrated benefits for pregnant women or care providers, high‐level evidence is scarce. High‐quality studies on innovations are needed to assess medical outcomes, patient and provider experiences and costs.

**Summary**

- What's already known about this topic?
  ◦ Digital health innovations in antenatal care are available to support or replace traditional provision of pregnancy care. In pregnancy ultrasonography, examples include tele‐ultrasound services, self‐operated ultrasound, and handheld devices.
- What does this study add?
  ◦ Results from 31 studies on innovations in ultrasound show promising results for application. Although most studies could demonstrate a benefit for pregnant women or health care providers, high‐level evidence is scarce.

## 1 Introduction

Increased access to mobile phones, wireless internet, video communication and development of portable devices have contributed to rapid innovations using telecommunications within health care [1, 2]. The use of any form of telecommunication to enhance traditional health care is called telemedicine. Forms of telemedicine with use of wireless devices in obstetric management have been described by a variety of studies such as maternal blood pressure monitoring and remote cardiotocography. Possible advantages include improved patient satisfaction and engagement, fewer hospital visits, reduction of costs and provision of remote healthcare in low‐ and middle‐income countries [3, 4]. Particularly, the COVID‐19 pandemic had a profound influence on the urgent need for innovation in health care provision.

Ultrasound is the main imaging modality used to evaluate the fetal condition during pregnancy. Digital innovations can be used to support or replace traditional provision of pregnancy ultrasonography, using tele‐ultrasound or portable devices. Tele‐ultrasound allows for real‐time fetal monitoring during remote consultations, self‐operated ultrasound measurements by pregnant women and furthermore it provides opportunities for remote healthcare in low‐ and middle‐income countries. Tele‐ultrasound could have the opportunity to fill in several health‐care gaps [5]. Besides possible benefits in a clinical role, tele‐ultrasound also can be used as an educational tool [6].

Furthermore, handheld devices for pregnancy ultrasound can be used by a clinicians to directly influence management decisions. Images from these devices can be reviewed by the performer of the ultrasound or by a consulted expert in a tele‐ultrasound (remote) setting. Potential benefits of innovative handheld devices with and without telemedicine consultations include its portability, ease of use and accessibility and may be especially useful in remote or resource‐limited areas [7].

Given the rapid development of innovations in digital health, we aimed to provide an overview of the existing literature investigating the impact of tele‐ultrasound, handheld devices and self‐operated ultrasound for various purposes. The review will assess the applicability, feasibility, (dis)advantages and (clinical) outcomes of these innovations in pregnancy ultrasound.

## 2 Methods

### 2.1 Eligibility Criteria, Information Sources, Search Strategy

For this review, PRISMA guidelines were followed. A systematic literature search was performed in PubMed and EMBASE in October 2023. The search included various synonyms for ultrasound use in pregnancy care, combined with telemedicine, point‐of‐care, portable or handheld devices and digital health (see Supplementary Material S1 for the search strategy) and related Mesh terms and Emtree/exploded terms. Screening and reviewing of abstracts and full articles was done independently by two authors (J.H. and S.K.) and discrepancies were discussed to reach final conclusions. Reference lists were screened for additional inclusions. All manuscripts were evaluated for eligibility for inclusion in this review.

All English‐written, prospective and retrospective studies or trials using tele‐ultrasound in pregnancy in human subjects were included. Included articles of interest reported clinical outcomes as well as outcomes related to experiences and costs.

### 2.2 Data Extraction and Analysis

Data were extracted by two independent researchers (J.H. and S.K.). Data extracted from each study included: author(s), publication year, study location, patient demographics, sample size, used ultrasound innovation or method (e.g., real‐time vs. asynchronous remote care), and outcome specific results. Indications for ultrasound were categorized into the following groups: first‐trimester routine scan (for viability or dating), first‐trimester anomaly scan, second‐trimester anomaly scan, ultrasound in high‐risk pregnancy after referral (e.g., fetal growth restriction [FGR], suspected fetal anomaly, multiple pregnancy), routine scan for fetal position, amniotic fluid and placental location, fetal biometry scan (with or without Doppler indices), transvaginal ultrasound (e.g., for cervical length). Also, the mean gestational age, sample size, ultrasound performer and ultrasound interpreter were extracted from all articles.

Risk of bias for each included study was assessed using the National Institutes of Health (NIH) Quality Assessment Tool for Observational Cohort and Cross‐Sectional Studies and the NIH Quality Assessment Tool for Controlled Intervention Studies. Both tools are designed to assist in the critical appraisal of study methodology and validity. Depending on the scores, studies were labeled good, fair, or low quality.

## 3 Results

### 3.1 Study Selection

The systematic literature search resulted in 435 articles (see Supplementary Material S2: Figure S1 PRISMA flow diagram). After removal of 177 duplicates and further screening of abstracts and full texts, 31 publications were included.

### 3.2 Study Characteristics and Quality Assessment

All articles were published between 1999 and 2023, with a peak in publications in 2020–2023. Two studies were controlled intervention studies, the remaining articles were cohort or cross‐sectional studies, and one qualitative study.

**FIGURE 1:**
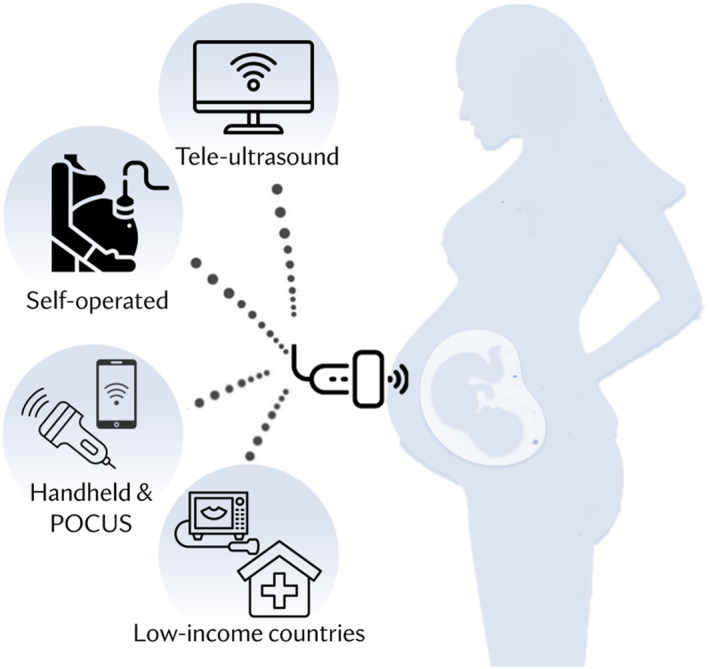
Telehealth innovations in four domains of ultrasound in pregnancy care.

According to the category of indication for ultrasound, one out of 31 studies studied innovations in viability scans, five out of 31 during second‐trimester anomaly scans, five articles on ultrasound in high‐risk pregnancy after referral (e.g., for FGR or suspected fetal anomaly), 18 articles on routine scans, and seven articles on fetal biometry. No articles were identified that studied tele‐ultrasound innovations in the first trimester anomaly scan or use of transvaginal ultrasound (e.g., for cervical length). The included articles were categorized into four domains, which will be addressed in the following paragraphs: tele‐ultrasound services (including 11 studies), patient‐operated ultrasound (three studies), handheld ultrasound devices (eight studies) and Low and middle‐income countries (nine studies) (Figure 1). Outcomes regarding costs of care were mentioned in five studies. Details from the results on experiences of either care providers or patients were summarized separately.

In Tables 1, 2, 3, 4, an overview of all selected studies is given in the four domains in which tele‐ultrasound in pregnancy care was described, implemented, or compared with standard care.

**TABLE 1:** Tele‐ultrasound services: overview of literature.

Author, year	Title article	Intervention location	Study design	Sample size	Indication of ultrasound	Mean gestational age (weeks)	Ultrasound performer	Ultrasound interpreter	Tele‐monitored (real‐time)
Whittington (2022) [8]	Detection of fetal anomalies by remotely directed and interpreted ultrasound (teleultrasound): A randomized noninferiority trial	United States	Randomized noninferiority trial	585 total, 294 interventions and 291 controls	Second trimester anomaly scan (because of increased risk of fetal anomaly)	22	Registered diagnostic medical sonographers	Maternal‐fetal medicine physicians	No
Smith (2021) [9]	Implementation of a fetal ultrasound telemedicine service: Women's views and family costs	United Kingdom	Cross‐sectional study	40	Ultrasound in high risk pregnancy after referral	28	Experienced sonographers	Fetal medicine specialists	Yes
Ferreira (2015) [10]	Trans‐Pacific tele‐ultrasound image transmission of fetal central nervous system structures	Brazil, Australia	Cross‐sectional study	15	Second trimester anomaly scan (including central nervous system scan)	24	1 experienced sonographer in Brazil	Fetal medicine specialists in Australia	No
Smith (2002) [11]	Tele‐ultrasound for remote areas	Scotland	Cross‐sectional study	85 video conferences	NR	NR	Sonographer in remote hospital	Fetal medicine unit	No
McCrossan (2011) [12]	Fetal diagnosis of congenital heart disease by telemedicine	Ireland	Prospective cohort	69	Ultrasound in high risk pregnancy after referral	23	Sonographer in general hospital	Fetal cardiologist	Yes
Adams (2018) [13]	A crossover comparison of standard and telerobotic approaches to prenatal sonography	Canada	Prospective cohort	30	*N* = 10 for second trimester anomaly scan and *n* = 20 for a (limited) routine scan	23	Tele‐robotic system, installed by an assistant, operated by a sonographer	Radiologist	No
Chan (2001) [14]	Realtime fetal ultrasound by telemedicine in Queensland. A successful venture?	Australia	Prospective cohort	71	Ultrasound in high risk pregnancy after referral	NR	Sonographers under the direction of the subspecialist.	Fetal medicine specialists	Yes
Soong (2002) [15]	The fetal tele‐ultrasound project in Queensland.	Australia	Cross‐sectional study	120	NR	NR	Sonographers under the direction of the subspecialist.	Fetal medicine specialists	Yes
Arbeille (2005) [16]	Fetal tele‐echography using a robotic arm and a satellite link	Spain	Cohort	29	*N* = 20 for second trimester anomaly scan, *n* = 9 referral after growth restriction or preeclampsia	34	Tele‐robotic system, installed by an assistant, operated by an expert (radiologist, obstetrician or midwife)	Expert (radiologist, obstetrician or midwife)	Yes
Brown (2017) [17]	Successful fetal tele‐Echo at a small Regional hospital	United States	Retrospective cohort	75	Ultrasound in high risk pregnancy after referral	27	Senior sonographer	Pediatric cardiologist	No
Rabie (2019) [18]	Teleultrasound for pre‐natal diagnosis: A validation study	United States	Validation study with retrospective cohort	2368 interventions with 3145 comparisons	Second trimester anomaly scan	23	Sonographers	Maternal‐fetal medicine specialists	Yes

Abbreviation: NR: not reported.

**TABLE 2:** Patient‐operated ultrasound: overview of the literature.

Author, year	Title article	Intervention location	Study design	Sample size	Indication of ultrasound	Mean gestational age (weeks)	Ultrasound performer	Ultrasound interpreter	Tele‐monitored (real‐time)	Ultrasound type
Hadar (2022) [19]	Mobile self‐operated home ultrasound system for remote fetal assessment during pregnancy	Israel	Observational cohort	101 women, 1360 selfscans	Routine scan	24	Patients (GA 14 + 0 to 39 + 6)	Two professionals	No	INSTINCT (PulseNmore, Omer)
Pontones (2023) [20]	Feasibility and acceptance of self‐Guided mobile ultrasound among pregnant women in routine prenatal care	Germany	Observational cohort	46	Routine scan	24	Patients	One professional	No	INSTINCT (PulseNmore, Omer) & Butterfly iQ (Butterfly)
Nir (2023) [21]	Integrating technologies to provide comprehensive remote fetal surveillance: A prospective pilot study	Israel	Observational cohort	10	Routine scan	40–41	Patients	NR	Yes	PulseNmore, Omer

Abbreviation: GA: gestational age.

**TABLE 3:** Handheld ultrasound devices: overview of the literature.

Authors (year)	Title article	Intervention location	Study design	Sample size	Indication for ultrasound	Mean gestational age (weeks)	Ultrasound performer	Ultrasound interpreter	Tele‐monitored (real‐time)	Ultrasound type
Omere (2022) [22]	Randomized trial of fundal height versus point‐of‐care ultrasound during routine antenatal visits	United States	Randomized controlled trial	138 total, 67 interventions and 71 controls	Fetal biometry	NR	Obstetrical care provider	Same as performer	No	Fujifilm Sonosite
Knights (2023) [23]	Impact of point‐of‐care ultrasound and routine third trimester ultrasound on undiagnosed breech presentation and perinatal outcomes: An observational multicentre cohort study	United Kingdom	Before‐after study	> 10,000	Routine scan	> 36 week	Health care provider	Same as performer	No	Vscan (GE)
Sayasneh (2012) [24]	Do pocket‐sized ultrasound machines have the potential to be used as a tool to triage patients in obstetrics and gynecology?	United Kingdom	Observational cohort	53	Routine scan	NR	Sonographers and physicians with low‐high experience	Same as performer	No	Vscan (GE)
Skendi (2022) [25]	Intrauterine pregnancy detection and gestational age assessment during Early pregnancy by a handheld point‐of‐care ultrasound device compared to a high‐End ultrasound system. An accuracy and Reliability study	France	Observational cohort	57	Viability scan	7	General practioner	Same as performer	No	Visiq (Philips)
Galjaard (2014) [26]	Use of a pocket‐sized ultrasound machine (PUM) for routine examinations in the third trimester of pregnancy	Belgium	Observational cohort	51	Routine scan	31	Experienced sonographer	Same as performer	No	Vscan (GE)
Leggett (2022) [27]	Incorporating personal‐device‐based point‐of‐care ultrasound into obstetric care: a validation study	United States	Observational cohort	100	Routine scan and/or fetal biometry	28	Sonographers or physicians	Same as performer	No	Butterfly iQ
Haragan (2015) [28]	Diagnostic accuracy of fundal height and handheld ultrasound‐measured abdominal circumference to screen for fetal growth abnormalities	United States	Observational cohort	251	Fetal biometry	32	Resident or specialist	Same as performer	No	Vscan (GE)
Corroenne (2023) [29]	Physicians' perceptions of the daily use of a handheld ultrasound device in the labor room	France	Cross‐sectional study	6	Routine scan	NR	Residents	Same as performer	NO	Vscan (GE)

Abbreviations: GA: gestational age; GE: general electric; NR: not reported.

**TABLE 4:** Low‐ and middle income countries: overview of the literature.

Authors (year)	Title article	Intervention location	Study design	Sample size	Indication for ultrasound	Mean gestational age	Ultrasound performer	Ultrasound interpreter	Tele‐monitored (real time)	Ultrasound type
Kozuki (2016) [30]	Accuracy of home‐based Ultrasonographic diagnosis of obstetric risk factors by primary‐level health workers in rural	Nepal	Cohort	804	Routine scan	NR (> 32 week)	Nurse midwives at home	Radiologist, obstetricians and sonographers	No	Sonosite nanomaxx portable ultrasound system
	Nepal									
Amoah (2016) [31]	Boosting antenatal care attendance and number of hospital deliveries among pregnant women in rural communities: A community initiative in Ghana based on mobile phones applications and portable ultrasound scans	Ghana	Cohort	122	Fetal biometry and routine scan	NR	Health care worker at home	Gynecologist in urban hospital	No	DP‐20, Mindray
Jemal (2022) [32]	Implementation and evaluation of a pilot antenatal ultrasound imaging programme using tele‐ultrasound in Ethiopia	Ethiopia	Cross‐sectional study	100	Routine antenatal care	NR	Health care provider	Obstetrician	Yes	Lumify
Vinayak (2017) [33]	Training midwives to perform Basic obstetric point‐of‐care ultrasound in rural areas using a tablet platform and mobile phone transmission Technology‐A WFUMB COE project	Kenya	Cohort	246	Fetal biometry and routine scan	NR	Midwives	Radiologists	No	VISIQ (Philips)
Vyas (2018) [34]	Feasibility study of minimally trained medical students using the rural Obstetrical ultrasound triage Exam (ROUTE) in rural Panama	Panama	Cohort	60	Fetal biometry and routine scan	> 14 week	Eight first year medical students from the United States	Obstetrician	No	Curvilinear probe (c60) with a Sonosite M‐turbo ultrasound machine
Dougherty (2021) [35]	Validation of a telemedicine quality assurance method for point‐of‐care obstetric ultrasound used in low‐resource settings	United States	Cohort	113	Routine scan	> 14–26 week	Sonographer	MFM specialist or radiologist	No	Voluson E8 system (GE)
							Medical student	MFM specialist, or radiologist		GE LOGIQ I system
Kodaira (2021) [36]	Reliability of ultrasound findings acquired with handheld apparatuses to inform urgent obstetric diagnosis in a high‐volume resource‐limited setting	Sierra Leone	Prospective observational cohort study	307	Routine scan	< 14 –37 week	House officers (intern junior doctors) and medical officers (resident doctors in training)	Experienced ultrasound operators	No	A US‐304 convex probe with 64 elements Lequio Power technology)
Toscano (2021) [37]	Testing telediagnostic obstetric ultrasound in Peru: a New horizon in expanding access to prenatal ultrasound	Peru	Feasibility study	126	Fetal biometry and routine scan	> 14 week	Nurse and care technician	Experienced radiologist	No	Portable Mindray DP‐10 (Mindray, China)
Kim (2023) [38]	Perceptions of service providers, service recipients and female community health volunteers on a rural obstetric ultrasound program in rural Nepal: a Qualitative study	Nepal	Qualitative study	93	Routine scan	NR	Nurses or midwives	Same as performer	No	NR

Abbreviations: GE: general electric; MFM: maternal fetal medicine; NR: not reported.

The quality of included (observational) cohort and cross‐sectional studies was generally low or fair based on the NIH quality assessment tool. Of the two controlled intervention studies, one was rated fair, and one was rated low quality. Detailed information can be found in Supplementary Material S3 (Table S1) and S4. Given the low or fair quality of studies and the heterogeneity/variety in study methods, the used technologies, and their outcome measurements, results were analyzed and presented in a narrative approach.

### 3.3 Tele‐Ultrasound Services

In 11 studies, the use of fetal tele‐ultrasound services, with or without real‐time expert evaluation, in the perinatal period was described (Table 1). All articles described the context of telemedicine between two different locations: one where the ultrasound is performed and assessed in the presence of the pregnant patient and one where the obtained images are reviewed by an expert in another location. Examples include the patient and sonographer being in a community clinic and the expert in a hospital, or the patient in a hospital and the expert in a tertiary referral clinic. If needed, results are to be discussed with the patient and/or ultrasound performer through videocalls, permitting real‐time consultations. In 5/11 studies, tele‐ultrasound services for the second trimester anomaly scan were examined [8, 10, 13, 16, 18] and 5/11 studies described telemedicine after referral for suspected (cardiac) anomalies, fetal growth restriction, or multiple pregnancy [9, 12, 14, 16, 17]. One study used telemedicine for routine ultrasound scans [13].

For the screening of anomalies in the second trimester, two studies found that tele‐ultrasound (by registered sonographers and reviewed by experts) proved to be non‐inferior to onsite detection of major fetal anomalies [8, 18]. The non‐inferiority randomized trial by Whittington et al. randomized 600 pregnant women for a second trimester anomaly scan to either in‐person visits versus tele‐ultrasound service with remote interpretation by an expert. With sensitivity of anomaly detection of 82 versus 85%, tele‐ultrasound was non‐inferior to in‐person visits [8].

Multiple studies found that tele‐ultrasound allows for secure diagnosis in the second trimester after referral in cases of (suspected) fetal malformations, with imaging of good quality [9, 10, 12, 17]. For example, a study from 2011 demonstrated that diagnosis of congenital heart anomaly was accurate in 97% of cases using tele‐ultrasound, after a standard in‐person visit in the reference center via a telemedicine link with live guidance by a fetal cardiologist [12].

Arbeille et al. studied a method to conduct fetal ultrasound using a robotic arm remotely controlled by an expert (tele‐echography). Visualization of fetal presentation, placental localization, echogenicity and amniotic fluid after positioning of the probe by an inexperienced assistant was good. In another study from 2018 using a robot, comparable results were found, however not all fetal biometry values were as accurate using the robot [13]. The absence of an expert on the patient side made specific measurements impossible, and therefore, the added benefit of robotic use remains doubtful [16].

Two studies mentioned effects on costs, of which two found cost savings for traveling costs of patients [9, 14].

### 3.4 Patient‐Operated Ultrasound

We found three studies that assessed self‐operated ultrasound in routine scans in pregnancy (Table 2).

In the study by Hadar et al, one hundred pregnant women were given a portable abdominal ultrasound device for self‐use for several scans a day for 1–2 weeks. Mean GA was 24 weeks ranging from 16 to 40 weeks. Images were stored and scored by two independent experts after completion of the study. The success rate in identifying several standard obstetric ultrasound parameters such as fetal heart activity, amniotic fluid volume and fetal body movements was high. The system was found to be a feasible solution for remote sonographic fetal assessment through self‐measurements [19].

In another study, 46 women were asked to self‐operate a mobile ultrasound device for one self‐assessment. Success rates of this first session varied from 52% to obtain a video of the fetal heartbeat and/or amniotic fluid, to 14% for a fetal profile image. The majority of the women would prefer live support by a physician during self‐examinations [20]. A pilot study for telemedicine visits for 40 + 1 to 40 + 6 weeks of gestation for fetal surveillance included self‐operated ultrasound for amniotic fluid assessment. Of 10 participants, all were able to obtain a satisfactory deepest vertical pocket after instructions [21].

None of the studies mentioned effects on healthcare costs.

### 3.5 Handheld Ultrasound Devices

Table 3 describes the eight studies that reported on the use of handheld ultrasound devices.

With increased portability, these innovative designs could also be of use in pregnancy ultrasound [39].

One out of eight included studies used handheld devices for viability scans, two for fetal biometry, four for routine scans, and one for both routine scans and biometry. One study used a personal‐device based point‐of‐care probe: a small portable ultrasound probe connected to a personal device such as smartphones and tablets. One‐hundred participants underwent fetal assessment using a standard ultrasound machine and a portable device. Among other things, estimated fetal weight, fetal biometry and detection of cardiac activity were assessed and showed near‐perfect agreement [27]. Similar findings were reported in two other studies and therefore, pocket‐sized ultrasound can be used for routine assessment of fetal growth and fetal wellbeing [24, 40].

Two studies evaluated whether screening of fetal growth using handheld devices instead of symphysis to fundal height (SFH) measurement could decrease the referral for a formal ultrasound. All ultrasound performers were trained to measure with a handheld device as well as SFH measurement. One study concluded that handheld ultrasound devices were a valuable screening tool as bedside fetal biometry measurements were comparable to standard ultrasound [28]. However, one study among low‐risk pregnant women found that it could not outperform SFH measurements as a screening tool for fetal growth [22]. A before‐after study showed that after implementation of routine scans with a handheld device in the third trimester (or before delivery), the proportion of undiagnosed breech presentations was reduced [23].

No studies were found that used handheld devices combined with telemedicine or tele‐ultrasound transmission of images between the ultrasound location and the other location of an expert. However, the three studies mentioned in paragraph of Patient‐operated ultrasound do use this modality.

None of these eight studies evaluated the effects on costs of healthcare during their research.

### 3.6 Low‐ and Middle‐Income Countries

In nine studies, the use of ultrasound innovations in low‐ and middle‐income countries was described (Table 4).

All eight studies described (tele‐)ultrasound innovations in second and third trimesters for routine scans or included fetal biometric parameters. Two of the eight studies used standardized obstetric sweep protocols performed by inexperienced health care providers for validation using routine ultrasound [35, 37].

In two studies, trained technicians acquired ultrasound scans using a portable ultrasound device and sent them to an expert (gynecologist). Ultrasound examinations were carried out for women who could not attend hospital visits for antenatal care. The combination of an online management system and tele‐ultrasound gave the possibility to report negative obstetric signs and therefore increased access to care in rural communities [31, 32].

Multiple studies have shown that health care workers with limited training can accurately diagnose selective obstetric risk factors using ultrasound. Kozuki et al. investigated the feasibility of ultrasound examinations performed by nurse‐midwives using handheld ultrasounds for the diagnosis of risk factors as non‐cephalic presentation, multiple gestation and placenta previa. The ultrasound images were reviewed by experts. Sensitivity, specificity, and positive and negative predictive values of the selected obstetric risk factors were high (between 90% and 100%). Therefore, hand‐held ultrasonography with limited training can accurately diagnose selected obstetric complications [30, 33, 34].

Another study assessed standardized obstetric sweeps taken with handheld devices by minimally trained medical students versus standard care obstetric ultrasound. Compressed movie clip ultrasound images were read and interpreted remotely by multiple experts and compared with the report of the ultrasound performer. The inter‐observer agreement for placental localization, fetal number, fetal presentation and pregnancy dating was high (Cohen *k* > 0.79). Parameters for fetal biometry using the sweeps were more difficult to obtain and interpret [35].

The limited human resources lead to task shifting or redistributing simple but effective tasks to less specialized health care workers with supervision of remote experts [30, 31]. Novel telediagnostic ultrasound systems and the expanding synergy between tele‐health and ultrasound can increase access to prenatal ultrasound imaging worldwide and are likely to enhance care and improve maternal and child health [37, 41].

In two studies, cost savings were mentioned with regard to reduced traveling costs for patients [32, 38].

### 3.7 Patient and Caregiver Experiences

Ten of the 31 aforementioned studies evaluated and reported patients' satisfaction and experiences with ultrasound innovations.

From the patients' perspective, studies on remote antenatal consultations reported that a main advantage was the access to a specialist unit without having to travel [11]. Overall, women expressed high levels of satisfaction with tele‐ultrasound and are willing to use the tele‐ultrasound service again and recommend it to others [8, 9, 15].

Hadar et al. studied the use of a self‐operated home ultrasound system for remote fetal assessment during pregnancy and reported that the user's satisfaction was high and the user's experience was scored with a 4.4 (± 0.6 in a range of 1–5).

Two studies from Kenya and Ethiopia described that patients felt the process was safe, convenient and reassuring; they all had a better antenatal visit experience and increased confidence in the delivery of care [32, 33].

The experiences and opinions of caregivers on the use of tele‐ultrasound or handheld devices have not been widely described. One study performed by Smith et al. in 2002 mentioned that the staff were uncomfortable about using the equipment but soon learned to use it confidently. One of the members of staff described it [tele‐ultrasound] as “almost as easy as using a telephone.” Another study by Smith reported that managers expressed concerns relating to the increased need for sonography and midwifery staff time to support the tele‐ultrasound service [9]. This was counterbalanced by the opportunity to increase sonographer skill levels and substantially reduce travel and associated costs for women and families. Six residents in obstetrics used a handheld ultrasound device in the labor ward and all were satisfied with its use and image quality [29]. A study from 2011 reported a high level of satisfaction amongst sonographers, feeling improvement in their skills and confidence during the research period [12]. A questionnaire about the implementation of tele‐ultrasound in Ethiopia described very positive reviews from users about the training, usability, and future role of telemedicine in patient care [32].

## 4 Discussion

### 4.1 Principal Findings

By providing this overview of the literature, we aimed to assess the applicability, feasibility, (dis)advantages and (clinical) outcomes of this innovative use of pregnancy tele‐ultrasound and handheld devices. This study reviewed the research in this field in four different categories. Most studies could demonstrate a benefit for pregnant women or health care providers; however, a high level of evidence from studies such as controlled trials is scarce.

In research with fetal tele‐ultrasound services, multiple studies have reported the feasibility and implementation of remote care. Three studies with patient‐operated ultrasound in pregnancy using self‐measurements concluded its feasibility with good‐to‐high patient experiences. Several studies have reported the results of the use of handheld ultrasound devices in pregnancy: multiple devices showed comparable results of analysis of pregnancy parameters including fetal growth. In low‐ and middle‐income countries, innovative use of ultrasound in pregnancy can facilitate pregnancy care provision. Ultrasound can be performed by trained as well as unskilled caregivers combined with remote evaluation by an expert. Studies show good accuracy of obstetric parameters and improvement of access to care in rural settings.

Effects of ultrasound innovations on medical or organizational costs were not available, besides the mention of cost savings with regard to traveling costs for patients.

Overall, 10 of 31 studies evaluated patient and caregiver experiences with tele‐ultrasound settings, most of them with good results amongst users.

### 4.2 Comparison With Previous Literature

Previous literature reviews have mentioned the use of innovations in pregnancy ultrasound while providing an update on digital health in pregnancy care [2, 3, 4, 39] Most of those mentioned studies on ultrasound were published before 2012 and demonstrated the usability of tele‐ultrasound services with respect to accuracy and acceptability amongst users. Our review combined the most recent studies on this topic, with most studies published in 2020–2023.

### 4.3 Study Limitations and Strengths

This overview of the literature has some limitations. Due to the heterogeneity of the found studies, the review was written in a narrative approach.

Strengths of this study included its extensive search strategy in multiple sources with the addition of reference screening and gray literature. As a result, we aimed to detect all relevant studies to review in this overview. In this narrative review, both medical as well as important secondary outcomes, such as experiences and costs, are described to provide a comprehensive overview of telemedicine innovations in ultrasound in pregnancy.

### 4.4 Implications and Conclusion

Overall, the results of this study on (tele‐)ultrasound innovations fit in the growing body of evidence for remote care in pregnancy monitoring, such as blood pressure self‐monitoring or remote cardiotocography [42, 43]. Advantages and disadvantages of tele‐ultrasound in pregnancy care are summarized in Table 5. Also, there are several indistinct areas of results that are equally important to rationalize its future use. In this rapidly evolving field of technology, future research should attain all domains of quality‐of‐care evaluation [44]. This includes safety of care, efficiency, patient‐centeredness as well as costs analysis. Regarding safety of care, future studies should compare innovative versus conventional ultrasound services in controlled of real‐world data studies to evaluate effects on medical outcomes of perinatal care. Future use by health care providers can be evaluated by qualitative or interview studies. Moreover, direct involvement of professionals in the development of future ultrasound innovations and services can aid in the uptake and implementation of innovations. Finally, costs of care should be taken into consideration. Introduction of remote ultrasound services could decrease costs by decreasing travel expenses or referral costs to tertiary care centers. However, the costs of the development and implementation of these innovations need to be balanced against the possible cost‐saving aspects.

**TABLE 5:** Advantages and disadvantages of current evidence in tele‐ultrasound innovations.

Tele‐ultrasound services	Patient operated ultrasound	Handheld ultrasound devices	Low and middle income countries
Advantages			
Increased access to care	Increased access to care	Increased access to care	Increased access to care
Good image quality	Use of mobile devices	Good image quality	Use of mobile devices
Detection of anomalies	Good patient experiences	Use of mobile devices	Task shifting with remote evaluation
Good patient experiences		Care providers experiences	Good patient experiences
			Care providers experiences
Disadvantages			
Limited data from controlled studies	Limited data from controlled studies	Limited data from controlled studies	Limited data from controlled studies
Indistinct			
Care providers experiences	Impact on costs	Patient experiences	Detection of anomalies
Impact on costs		Impact on costs	Impact on costs

Future directions of studies may focus on the combination of handheld ultrasound devices and remote interpretation by experts via tele‐ultrasound. In an outpatient setting, this combination can aid in triage and diagnosis of pregnancy complications in antenatal care with limited resources. Viewpoints of caregivers and patients should be part of evaluation studies for successful implementation in case of satisfactory results. Studies on patient‐operated ultrasound (from home) can research the possibilities and indications of this innovation, including the barriers and facilitators of mentoring patients in this technique. The use of (patient‐operated) obstetric sweep protocols is now under research to detect ultrasound anomalies or pregnancy complications [45]. These novel possibilities of tracking fetal parameters and artificial intelligence may aid in the follow‐up of complicated pregnancies with FGR or fetal anomalies.

## 5 Conclusions

This review has shown the available value of telehealth innovations for ultrasonography in pregnancy care. Several advantages and disadvantages have been found that influence future care. Study outcomes of high‐level evidence studies are scarce. Despite possible challenges for implementation, tele‐ultrasound is very likely to disperse globally as a result of progress in digital health care. Particularly, the COVID‐19 pandemic had a profound influence on the urgent need for innovation in health care provision. Future—high‐quality‐ research should focus on medical outcomes as well as patient experiences and costs to further evaluate health care innovation in pregnancy monitoring.

## Ethics Statement

The authors have nothing to report.

## Consent

The authors have nothing to report.

## Conflicts of Interest

The authors declare no conflicts of interest.

## Supporting information

Supporting Information S1

## Data Availability

The authors have nothing to report.
