# Supplementary material for: The Potential of Tele‐Ultrasound, Handheld and Self‐Operated Ultrasound in Pregnancy Care: A Systematic Review
Source: Prenat Diagn. 2024 Oct 10;45(7):906–20. doi: 10.1002/pd.6679 (PMC12180574; doi:10.1002/pd.6679)
Supplement: Supplementary file 1 — Supporting Information S1 [file PD-45-906-s001.docx]

**Supplementary material
S1. Search strategy
S2. Figure S1. PRISMA Flow diagram
S3. Table S1. Risk of bias assessment
S4. Questions of the NIH Quality Assessment Tools**

**S1. Search Strategy**

**Pubmed**

| **Tele/remote/Mobile** | **("tele-medicine"[Title/Abstract] OR "tele-medicine"[Title/Abstract] OR "mobile"[Title/Abstract] OR "self-operated"[Title/Abstract] OR "self-operated"[Title/Abstract] OR "tele-monitoring"[Title/Abstract] OR "tele-monitoring"[Title/Abstract] OR "tele-ultrasound"[Title/Abstract] OR "E-health"[Title/Abstract] OR "Ehealth"[Title/Abstract] OR "Mhealth"[Title/Abstract] OR "M-health"[Title/Abstract] OR "mobile ultrasound"[Title/Abstract] OR "home ultraso*"[Title/Abstract] OR "tele-monitor*"[Title/Abstract] OR "tele ultraso*"[Title/Abstract] OR "tele-ultraso*"[Title/Abstract]) OR "point of care ultrasound"[Title/Abstract] OR "POCUS"[Title/Abstract] OR "handheld"[Title/Abstract]** |
| --- | --- |
| **Ultrasound** | **AND ("ultrasound"[MeSH Terms] OR "ultrasonography"[MeSH Terms] OR "ultrasonics"[MeSH Terms] OR "ultraso*"[Title/Abstract])** |
| **During pregnancy** | **AND (“Pregnancy”[Mesh] OR “Pregnan*”[tiab] OR "Fetus"[Mesh] OR "Fetus*"[tiab] OR "Foetus*"[tiab] OR "Foetal"[tiab] OR "Fetal"[tiab] OR "prenatal*"[tiab] OR "antenatal*"[tiab] OR "pre natal*"[tiab] OR "ante natal*"[tiab] OR "in utero"[tiab] OR "intrauterine"[tiab] OR "intra uterine"[tiab])** |

**Embase search**

| **Tele/remote/Mobile** | **('tele-medicine':ti,ab,kw OR 'mobile':ti,ab,kw OR 'self-operated':ti,ab,kw OR 'tele-monitoring':ti,ab,kw OR 'telemonitoring':ti,ab,kw OR 'tele-ultrasound':ti,ab,kw OR 'e-health':ti,ab,kw OR 'ehealth':ti,ab,kw OR 'mhealth':ti,ab,kw OR 'm-health':ti,ab,kw OR 'mobile ultrasound':ti,ab,kw OR 'home ultraso*':ti,ab,kw OR 'tele-monitor*':ti,ab,kw OR 'tele ultraso*':ti,ab,kw OR 'tele-ultraso*':ti,ab,kw) AND** |
| --- | --- |
| **Ultrasound** | **('echography'/exp OR echography OR 'echography':ti,ab,kw OR 'ultrasound'/exp OR ultrasound OR 'ultraso*':ti,ab,kw OR 'ultrasound':ti,ab,kw) AND** |
| **During pregnancy** | **('fetus'/exp OR fetus OR 'fetus*':ti,ab,kw OR 'foetus*':ti,ab,kw OR 'foetal':ti,ab,kw OR 'fetal':ti,ab,kw OR 'prenatal'/exp OR prenatal OR 'prenatal*':ti,ab,kw OR 'antenatal*':ti,ab,kw OR 'pre natal*':ti,ab,kw OR 'ante natal*':ti,ab,kw OR 'in utero':ti,ab,kw OR 'intrauterine':ti,ab,kw OR 'intra uterine':ti,ab,kw)** |

**S2. Figure S1. PRISMA flow diagram**

Records identified through Pubmed search
(n = 329)

## Screening

## Included

## Eligibility

## Identification

Records identified through Embase search
(n = 106)

Records after combining searches
(n = 435)

Records screened [Title/Abstract]
(n = 258)

Records excluded based on in- and exclusion criteria*
(n = 227)

Full-text articles assessed for eligibility
(n = 31)

Full-text articles excluded:

- Not relevant due to domain: 7

Studies included in review
(n =24)

Duplicates excluded
(n = 177)

Snowballing reference list included articles (n=7)

Studies included in review
(n =31)

**S3. Table S1. Risk of bias assessment**

**
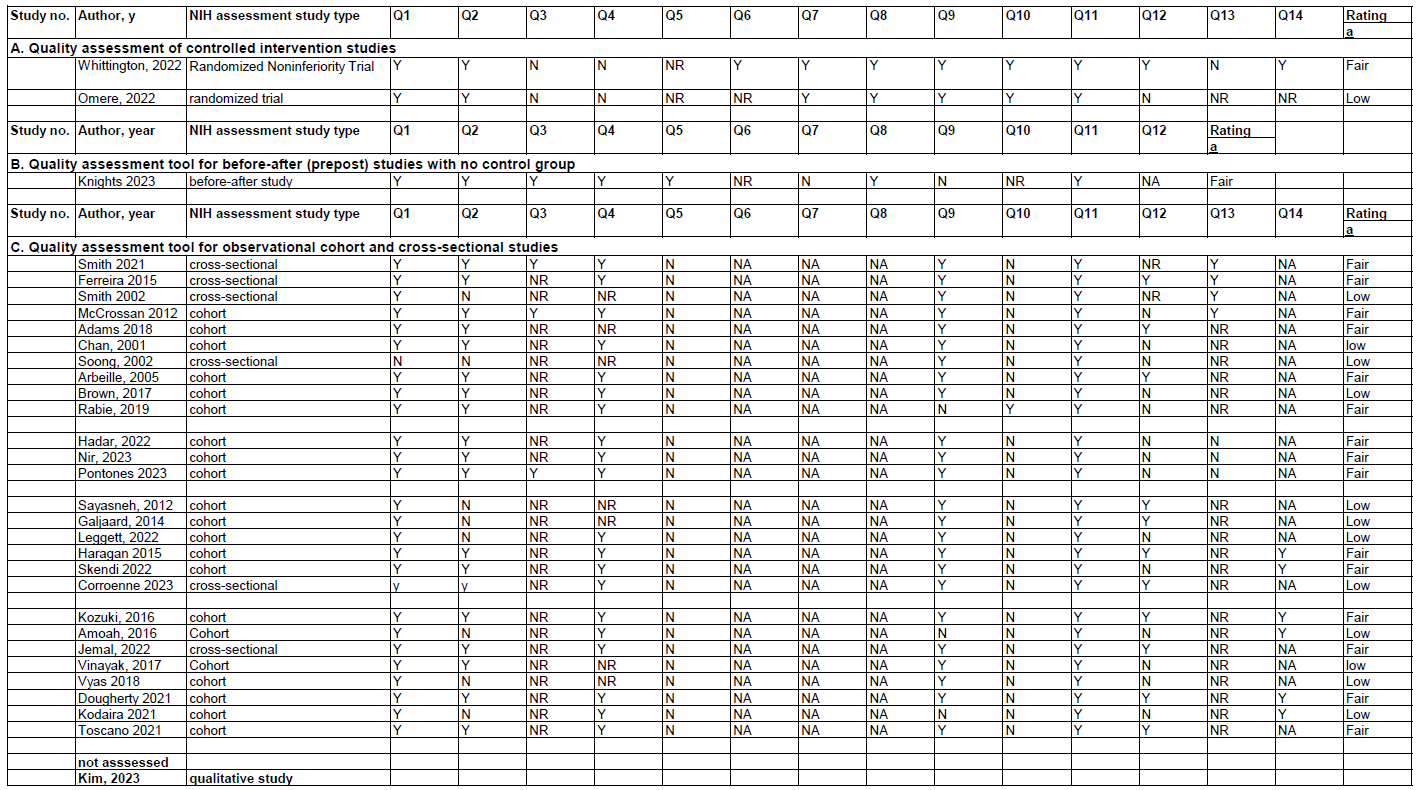
**

**S4. Questions of the NIH Quality Assessment Tools**

| **A. Quality assessment of controlled intervention studies** |
| --- |
| Q1. Was the study described as randomized, a randomized trial, a randomized clinical trial, or a randomized controlled trial? |
| Q2. Was the method of randomization adequate (ie, use of randomly generated assignment)? |
| Q3. Was the treatment allocation concealed (so that assignments could not be predicted)? |
| Q4. Were study participants and providers blinded to treatment group assignment? |
| Q5. Were the people assessing the outcomes blinded to the participants’ group assignments? |
| Q6. Were the groups similar at baseline on important characteristics that could affect outcomes (eg, demographics, risk factors, or comorbid conditions)? |
| Q7. Was the overall dropout rate from the study at endpoint ≤20% the number allocated to treatment? |
| Q8. Was the differential dropout rate (between treatment groups) at endpoint 15 percentage points or lower? |
| Q9. Was there high adherence to the intervention protocols for each treatment group? |
| Q10. Were other interventions avoided or similar in the groups (eg, similar background treatments)? |
| Q11. Were outcomes assessed using valid and reliable measures, implemented consistently across all study participants? |
| Q12. Did the authors report that the sample size was sufficiently large to be able to detect a difference in the main outcome between groups with at least 80% power? |
| Q13. Were outcomes reported or subgroups analyzed prespecified (ie, identified before analyses were conducted)? |
| Q14. Were all randomized participants analyzed in the group to which they were originally assigned (ie, did they use an intention-to-treat analysis)? |
| **B. Quality assessment tool for before-after (prepost) studies with no control group** |
| Q1. Was the study question or objective clearly stated? |
| Q2. Were eligibility and selection criteria for the study population prespecified and clearly described? |
| Q3. Were the participants in the study representative of those who would be eligible for the test, service, or intervention in the general or clinical population of interest? |
| Q4. Were all eligible participants that met the prespecified entry criteria enrolled? |
| Q5. Was the sample size sufficiently large to provide confidence in the findings? |
| Q6. Was the test, service, or intervention clearly described and delivered consistently across the study population? |
| Q7. Were the outcome measures prespecified, clearly defined, valid, reliable, and assessed consistently across all study participants? |
| Q8. Were the people assessing the outcomes blinded to the participants’ exposures or interventions? |
| Q9. Was the loss to follow-up after baseline ≤20%? Were those lost to follow-up accounted for in the analysis? |
| Q10. Did the statistical methods examine changes in outcome measures from before to after the intervention? Were statistical tests done that provided P values for the pre-to-post changes? |
| Q11. Were outcome measures of interest taken multiple times before the intervention and multiple times after the intervention (ie, did they use an interrupted time series design)? |
| Q12. If the intervention was performed at a group level (eg, a whole hospital or a community) did the statistical analysis take into account the use of individual-level data to determine effects at the group level? |
| **C. Quality assessment tool for observational cohort and cross-sectional studies** |
| Q1. Was the study question or objective clearly stated? |
| Q2. Was the study population clearly specified and defined? |
| Q3. Was the participation rate of eligible persons at least 50%? |
| Q4. Were all the subjects selected or recruited from the same or similar populations (including the same period)? Were inclusion and exclusion criteria for being in the study prespecified and applied uniformly to all participants? |
| Q5. Was a sample size justification, power description, or variance and effect estimates provided? |
| Q6. For the analyses in this article, were the exposures of interest measured before the outcomes being measured? |
| Q7. Was the timeframe sufficient so that one could reasonably expect to see an association between exposure and outcome if it existed? |
| Q8. For exposures that can vary in amount or level, did the study examine different levels of the exposure as related to the outcome (eg, categories of exposure or exposure measured as continuous variable)? |
| Q9. Were the exposure measures (independent variables) clearly defined, valid, reliable, and implemented consistently across all study participants? |
| Q10. Was the exposures assessed more than once over time? |
| Q11. Were the outcome measures (dependent variables) clearly defined, valid, reliable, and implemented consistently across all study participants? |
| Q12. Were the outcome assessors blinded to the exposure status of participants? |
| Q13. Was loss to follow-up after baseline ≤20%? |
| Q14. Were key potential confounding variables measured and adjusted statistically for their impact on the relationship between exposures and outcomes)? |
| **N, no; NA, not applicable; NIH, National Institute of Health; NR, not reported; Q, question; Y, yes.** |
| **a Of note, 3 ratings were possible: “good,” “fair,” or “poor.** |
